# Non-invasive assessment of glymphatic dysfunction in middle cerebral artery stenosis based on DTI-ALPS and ro-ALPS

**DOI:** 10.3389/fneur.2026.1826663

**Published:** 2026-06-17

**Authors:** Yidi Zhu, Luoyu Wang, Liqing Zhang, Xue Tang, Maoling Liu, Qingqing Wen, Darong Zhu, Han Zhang, Xiufang Xu, Zhongxiang Ding

**Affiliations:** 1 School of Medical Imaging, Hangzhou Medical College, Hangzhou, China; 2 School of Biomedical Engineering, ShanghaiTech University, Shanghai, China; 3 Department of Radiology, Affiliated Hangzhou First People’s Hospital, School of Medicine, Westlake University, Hangzhou, China; 4 MR Research, GE Healthcare, Beijing, China

**Keywords:** choroid plexus, cognitive dysfunction, diffusion tensor imaging along the perivascular space index, glymphatic system, middle cerebral artery stenosis

## Abstract

**Background:**

Middle cerebral artery stenosis (MCA-S) impairs cerebral perfusion and vascular pulsatility, potentially disrupting cerebrospinal fluid dynamics. We evaluated perivascular diffusivity in MCA-S using diffusion tensor image analysis along the perivascular space (DTI-ALPS) index and the redirected ALPS (ro-ALPS) index, and assessed their clinical correlations.

**Methods:**

Thirty-seven patients with MCA-S and 41 matched healthy controls (HCs) underwent MRI and cognitive function testing. We compared the DTI-ALPS index and ro-ALPS indices between the two groups and performed correlation analyses with cognitive scores and clinical indicators, including high-density lipoprotein, homocysteine, and choroid plexus (CP) volume.

**Results:**

The results showed that both DTI-ALPS index and ro-ALPS index were significantly lower in the MCA-S group than in the HC group (both *p* < 0.001). In MCA-S patients, the DTI-ALPS index was negatively correlated with CP volume (*r* = −0.541, *p_FDR_* = 0.012), and the ro-ALPS index showed a negative correlation with CP volume (*r* = −0.568, *p_FDR_* < 0.001).

**Conclusion:**

These findings suggest that perivascular clearance is impaired in patients with MCA-S, as reflected by lower ALPS indices. These ALPS indices, as imaging biomarkers, show significant associations with CP volume, suggesting their potential utility in monitoring disease progression.

## Introduction

Middle cerebral artery stenosis (MCA-S) is one of the most common sites of atherosclerotic stenosis in Asian populations (1–3). Pathologically, this process begins with endothelial damage, progresses through lipid-streak formation within the arterial wall, and leads to atheromatous plaque development (4, 5). As the disease progresses and the vessel wall is remodeled, plaque may gradually encroach on the vascular lumen and may eventually cause stenosis, plaque rupture or detachment, complete lumen occlusion, and acute cardiovascular or cerebrovascular events (6). Progressive vascular wall stiffening attenuates normal pressure and diameter fluctuations during the cardiac cycle, thereby reducing middle cerebral artery pulsatility; this attenuation in pulse-wave amplitude and velocity can be detected by transcranial Doppler ultrasound or four-dimensional flow magnetic resonance imaging (7, 8).

The glymphatic system is a perivascular network mediated by astrocytic aquaporin-4 (AQP4) channels and relies on arterial pulsations as a major ‘hydraulic’ driving force for cerebrospinal fluid (CSF) and interstitial fluid (ISF) exchange and metabolic waste clearance (9–11). Animal studies have shown that reduced arterial pulsatility directly compromises glymphatic flow (12). Thus, impaired MCA pulsatility provides a plausible mechanistic link between intracranial atherosclerosis and cerebral clearance dysfunction, although direct evidence in MCA-S patients remains limited.

Traditional assessment of glymphatic function relies on two-photon microscopy tracer tracking or MRI methods requiring intrathecal gadolinium contrast injection (10, 13–16). Thus, non-invasive methodological approaches are essential. Diffusion tensor imaging analysis along the perivascular space (DTI-ALPS), proposed by Taoka et al. (17) in 2017 provides a non-invasive surrogate measure of perivascular diffusivity related to glymphatic function. This technique exploits the anatomical orientation of projection and association fibers around the medullary veins in the lateral ventricular region and estimates CSF-ISF exchange efficiency by quantifying water diffusion along the perivascular direction. In this method, regions of interest (ROI) are placed in projection and association fibers adjacent to the medullary vein at the level of the lateral ventricular level. However, reliable ALPS measurement requires accurate ROI alignment relative to subject head position. Subsequent methodological improvements, including automated ROI localization and diffusion tensor reorientation, have been proposed to improve measurement reproducibility (18–20). In the present study, we adopted a reorientation technique that maps vector images into a standardized space and generates redirected diffusivity maps, defined as the reoriented ALPS (ro-ALPS) index (21). Reduced ALPS index has been reported in Alzheimer disease (22, 23), Parkinson disease (24), normal-pressure hydrocephalus (18), Moyamoya disease (25, 26), and acute ischemic stroke patients (27), suggesting that this metric is sensitive to multiple types of perivascular clearance impairment.

In this study, we jointly applied the conventional DTI-ALPS index and the ro-ALPS index methods to investigate the association between MCA-S and perivascular clearance impairment. In a prospective cohort, we aimed to (1) compare the ALPS indices in patients with unilateral MCA-S with age-, gender-, and education-matched HCs; and (2) examine associations between ALPS indices and the stenosis-related clinical variables, neurocognitive function, and CP volume.

## Materials and methods

### Participants

**Figure 1:**
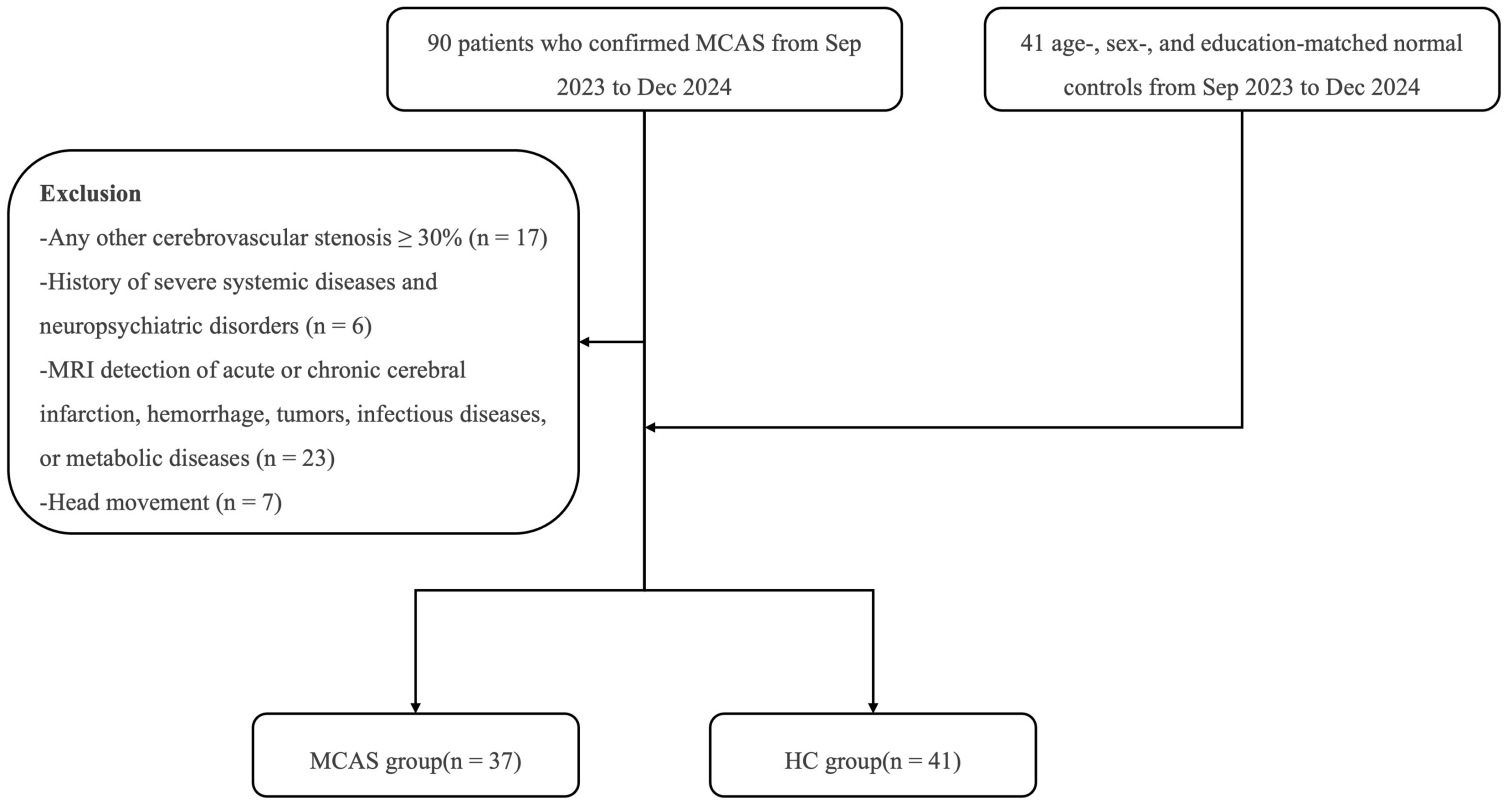
Flowchart of the present study.

This study included 37 patients with MCA-S who were recruited from the Department of Neurology at the First People’s Hospital of Hangzhou from September 2023 to December 2024, and 41 healthy controls (HCs) matched for age, sex, and education level who are recruited through the hospital health promotion center. The inclusion criteria: (1) age 18–80 years; (2) patients with unilateral MCA-S (luminal narrowing > 50%) confirmed by computed tomography angiography (CTA), magnetic resonance angiography (MRA), or digital subtraction angiography (DSA); and (3) right-handedness. The exclusion criteria: (1) neurological diseases such as cerebral aneurysm, brain tumor, traumatic brain injury, anxiety disorder, depression, or schizophrenia; (2) neurodegenerative diseases such as AD, PD, or epilepsy; (3) disorders that significantly affect cognitive assessment, such as language, hearing, or vision disorders; (4) a history of substance abuse, alcohol poisoning or carbon monoxide poisoning; (5) cerebral infarction, hemorrhage, or encephalomalacia visible on MRI that could interfere with DTI analysis; and (6) relative or absolute contraindications to MRI. During the study period, HCs were subject to the same exclusion criteria as MCA-S patients. This study was approved by the Ethics Committee of the Hangzhou First People’s Hospital (IIT-20230822-0181-01). All methods were conducted in accordance with the Declaration of Helsinki, and all participants provided written informed consent (Figure 1).

### Neuropsychological evaluation

All participants underwent comprehensive clinical psychological assessments administered by specialized physicians. Cognitive function was evaluated using the Mini-Mental State Examination (MMSE), while depressive and anxiety symptoms were assessed via the Self-Rating Depression Scale (SDS) and Self-Rating Anxiety Scale (SAS), respectively. Sleep quality was measured with the Pittsburgh Sleep Quality Index (PSQI). According to Chinese normative standards, the established cutoff scores were 50 for SAS (indicating clinical anxiety) and 53 for SDS (indicating clinical depression). For PSQI global scores: <5 indicates good sleep quality, 5–8 indicates poor sleep quality, and >8 indicates probable insomnia or severe sleep disturbances.

### MRI data acquisition

Each subject underwent a cranial MRI scan using a 3.0-T MRI scanner (SIGNA Premier, GE HealthCare, Waukesha, WI, United States) and a 48-channel phased-array head coil. During scanning, participants were positioned supine and head-first in the scanner. They were instructed to close their eyes, remain awake, breathe calmly, and avoid head motion (28, 29). Foam pads were placed on both sides of the head for stabilization, and earplugs were used to reduce noise and protect hearing. Scans were acquired between 1:00 p.m. and 8:00 p.m. to reduce inter-subject variability related to sleep status. The scanning parameters were as follows:

1) T1-weighted 3D fast spoiled gradient-recalled imaging (3D-T1WI): repetition time (TR) = 2,616 ms, echo time (TE) = 2.9 ms, inversion time (TI) = 1,000 ms, flip angle (FA) = 8°, matrix size = 256 × 256, field of view (FOV) = 256 mm × 256 mm, voxel size = 1 mm × 1 mm × 1 mm, slice thickness/gap = 1/1 mm, and 192 slices.
2) Axial diffusion-weighted imaging (DWI): TR = 3,600 ms, TE = 62 ms, matrix size = 128 × 128, FOV = 256 mm × 256 mm, voxel size = 2.0 mm × 2.0 mm × 2.0 mm, slice thickness/gap = 2/0 mm, 75 slices, and b-values = 0, 1,000, and 2000s/mm^2^. Although multi-shell data (b = 0, 1,000, and 2000 s/mm^2^) were acquired, only the b = 1,000 s/mm^2^ shell was used for DTI processing and ALPS index calculation in accordance with the standard methodology. We selected b = 1,000 s/mm^2^ to align with prior validation studies of DTI-ALPS, which demonstrate optimal sensitivity to perivascular fluid diffusion at this b-value (17, 19).

### MRI data processing

#### DTI data preprocessing

The DWI data were preprocessed using MRtrix3 (30) to ensure data quality (31). Key steps included: denoising, Gibbs ringing artifact correction, susceptibility-induced distortion correction, eddy-current correction, and B1 field inhomogeneity correction. Fractional anisotropy (FA) maps were subsequently generated using the dtifit tool from FSL (FMRIB Software Library v6.0) using only the b = 0 and *b* = 1,000 s/mm^2^ volumes.

### Region-of-interest (ROI) definition in native space

**Figure 2:**
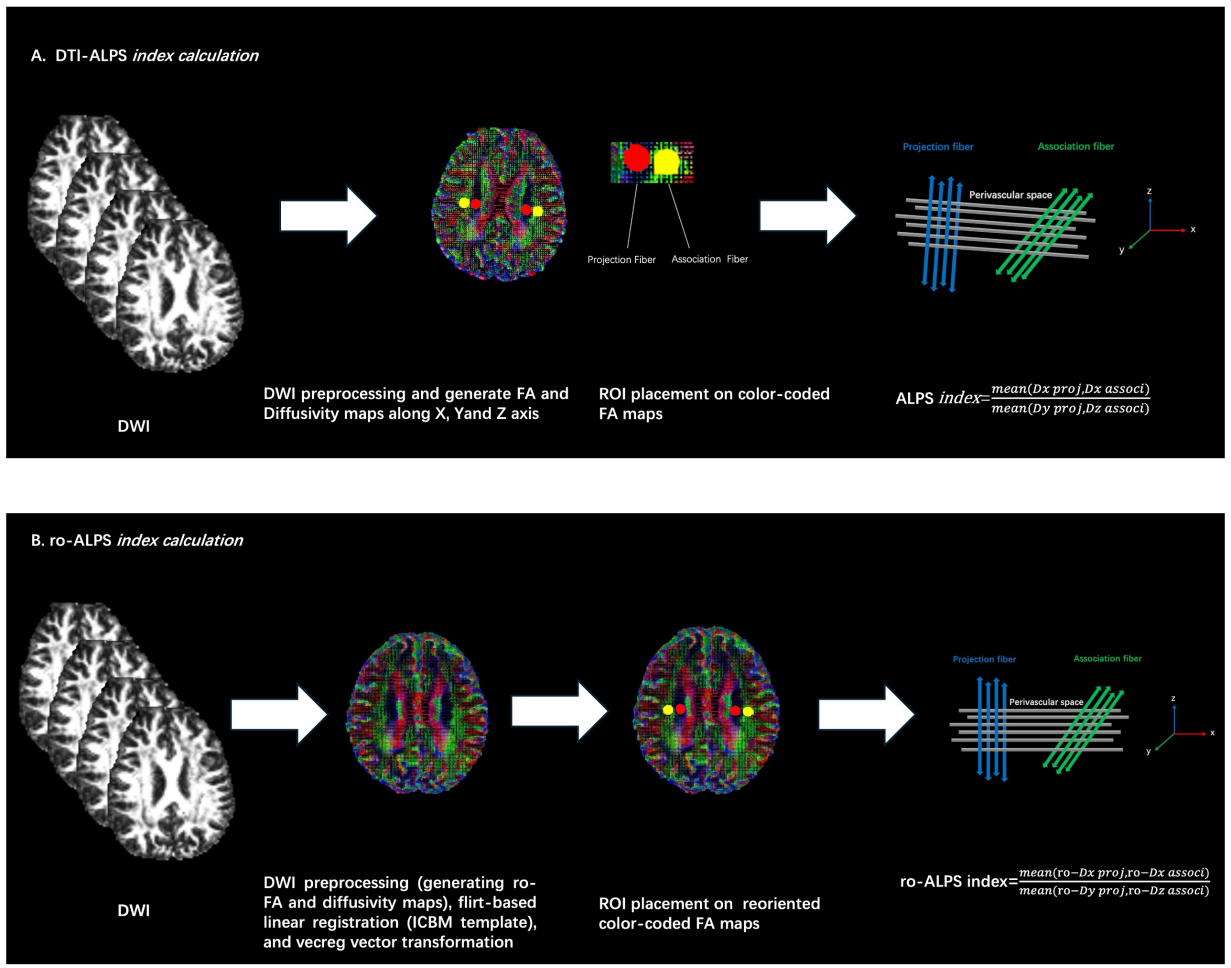
DTI-ALPS processing flowchart. This flowchart illustrates two methods for calculating the ALPS index using diffusion tensor imaging (DTI): the conventional DTI-ALPS index **(A)** and the optimized ro-ALPS index incorporating linear registration and vector reorientation **(B)**. Key steps include diffusion-weighted image (DWI) preprocessing, generation of fractional anisotropy (FA) and directional diffusivity maps, region of interest (ROI) placement on color-coded FA maps, and final index computation.

The ROIs were defined based on the JHU ICBM-DTI-81 White-Matter Labels Atlas^1^ (32). Projection fibers were represented by the corticospinal tract (CST), and association fibers were represented by the superior longitudinal fasciculus (SLF) (17, 19). The Montreal Neurological Institute (MNI) coordinates of the centers of the left and right ROIs were (24, −12, 24) and (−28, −12, 24) in projection fibers, respectively. The MNI coordinates of the centers of the left and right ROIs were (36, −12, 24) and (−40, −12, 24) in association fibers, respectively (19). Spherical masks with a 5 mm radius (Figure 2A) were centered on these coordinates to restrict analysis to fibers adjacent to the lateral ventricles (19). Individual FA maps were non-linearly registered to the ICBM FA template (1 mm isotropic) using Advanced Normalization Tools (ANTs), generating forward and inverse transformation fields. The standard MNI coordinates were inversely transformed into each subject’s native DTI space to ensure anatomically accurate ROI placement. All ROI placements were manually inspected by two senior neuroradiologists to ensure that they were located within normal-appearing white matter and were free from any focal infarction or signal abnormalities. Inter-rater reliability was assessed in a random subset of 30 participants using Cohen’s kappa coefficient, yielding a value of 0.93. Any discrepancies in ROI placement identified during this verification process were resolved by consensus between the two neuroradiologists.

### ALPS index calculation

The ALPS index for each cerebral hemisphere was calculated from the single-shell diffusion data (*b* = 1,000 s/mm^2^) following the original methodology (13). A mean ALPS index (mALPS) was computed as the average of the left and right hemispheric ALPS index. As shown below:

ALPS_index (left/right) = mean(Dx_proj, Dx_assoc)/mean(Dy_proj, Dz_assoc).

### Spatial reorientation

To enable standardized ROI placement for the ro-ALPS index, each subject’s ro-FA map was first linearly registered to the ICBM FA template using FSL flirt with 6 degrees of freedom (translation and rotation only, no scaling). The resulting transformation matrices were then applied to the vector data (diffusivity maps) using FSL vecreg tool (31, 33). This procedure generated realigned diffusivity maps oriented consistently within the ICBM space. Then, the diffusivity was recorded in the directions of the Dx, Dy, and Dz of ROIs on projection fibers and association fibers as ro-Dxproj, ro-Dyproj, ro-Dzproj, ro-Dxassoc, ro-Dyassoc, and ro-Dzassoc. Realigned color-coded FA maps (ro-FA) were also generated.

### Region-of-interest (ROI) definition in realigned space

The ro-FA maps were non-linearly registered to the ICBM FA template using ANTs (as in Step 2), generating new transformation fields. The same standard MNI coordinates used in Step 2 were then inversely transformed into the realigned space (ro-FA space) using the inverse ANTs transformation (Figure 2B). This ensured ROIs were placed in anatomically equivalent locations within the consistently oriented diffusivity data. ROI placement was neuroradiologically verified. Eigenvalues extracted from the realigned diffusivity maps within these ROIs were recorded as ro-Dx_proj, ro-Dy_proj, ro-Dz_proj, ro-Dx_assoc, ro-Dy_assoc, and ro-Dz_assoc.

### Ro-ALPS calculation

The ro-ALPS index for each hemisphere was calculated using the realigned diffusivity values (13, 31). A mean ro-ALPS index was computed as the average of the left and right hemispheric ro-ALPS index (Figure 2B). As shown below:

ro-ALPS_index (left/right) = mean(ro-Dx_proj, ro-Dx_assoc)/mean(ro-Dy_proj, ro-Dz_assoc).

### CP volume estimation

The segmentation of the choroid plexus (CP) was based on preprocessed T1-weighted images data using the deep learning model chp_seg v1.0.1 (GitHub open source code repository: https://github.com/Center-of-Imaging-Biomarker-Development/chp_seg) (34). All T1-weighted MRI images underwent realignment followed by N4 bias correction and AC-PC alignment. To ensure segmentation quality, all results were manually reviewed by senior radiologists, and errors were manually corrected. In addition, to reduce variability among subjects, CP volume was normalized by total ntracranial volume (TIV) as provided by SynthSeg.

### Statistical analysis

Statistical analysis was performed using SPSS (version 27.0 IBM Corporation) software. Normality was assessed using the Shapiro–Wilk test and visual assessment of histograms. Based on these results, normally distributed variables are presented as mean ± SD and were compared between groups using the independent-samples *t*-test. Non-normally distributed variables are presented as median (IQR) and were compared using the Mann–Whitney *U* tests. Categorical variables were compared using the chi-square tests. A *p*-value < 0.05 was considered statistically significant. We performed a general linear model (GLM) analysis to control for potential confounders, including age, sex, years of education, hypertension, and diabetes. For correlation analyses, although the DTI-ALPS index and ro-ALPS index were normally distributed, the neuropsychological scores (MMSE, MoCA, and SDS) were non-normally distributed. According to statistical principles, when at least one variable in a pair is non-normally distributed, a nonparametric test is appropriate. Therefore, we used two-tailed Spearman’s rank correlation to assess the relationships between the ALPS indices and clinical indicators, neuropsychological scores, and CP volume. All statistical tests were two-tailed, with significance set at *p* < 0.05. Adjustments for multiple comparisons were performed using the false discovery rate (FDR) method where indicated.

## Results

### Demographics and clinical characteristics

After excluding subjects who did not meet the enrollment criteria, we included 37 patients with MCA-S (mean age = 58.2 years, SD = 14.9 years; 22 males and 15 females) and 41 age- and sex-matched (all right-handed) HCs (mean age = 59.4 years, SD = 12.9 years, 22 males and 19 females). All participants were right-handed and completed clinical screening, neurocognitive assessment, and MRI acquisition. Table 1 demonstrates the basic clinical information and neuropsychological scores of the study subjects. Statistical analysis revealed significant differences in the prevalence of hypertension (*p* = 0.02), diabetes mellitus (*p* = 0.02), and MMSE scores (*p* = 0.01) between the two groups. Specifically, the MCA-S group had higher prevalences of hypertension and diabetes and lower MMSE scores than the HC group. No statistically significant between-group differences were observed in age, sex, years of education, BMI, TIV, MoCA scores, SAS scores, SDS scores, PSQI scores, smoking prevalence, drinking prevalence, hyperlipidemia, or coronary heart disease (all *p* > 0.05). Normality results are presented in the Supplementary Table S1. No missing data were present for variables included in the primary between-group analyses. HDL and homocysteine were available only in the MCA-S group and therefore were not included in the between-group comparisons. We have clarified this in Table 1 and in the Methods section.

**Table 1:** Demographic and clinical characteristics of MCA-S and HC groups.

Variable	*t*/χ^2^/*U*	MCA-S (*N* = 37)	HC (*N* = 41)	*p*-value
Gender (male: female)	0.27	22:15	22:19	0.61
Age (years)	742.50	60.00 (52.00, 70.50)	60.00 (52.50, 69.50)	0.87
Education (years)	656.00	8.00 (2.00, 9.00)	8.00 (5.00, 11.00)	0.30
BMI	612.00	24.44 (23.03, 26.56)	23.59 (22.00, 25.03)	0.14
TIV	0.31	1446.30 ± 156.91	1436.06 ± 136.49	0.76
MMSE	493.50	28.00 (23.00, 29.00)	29.00 (27.00, 29.50)	**0** .01*
MoCA	696.00	25.00 (20.00, 28.00)	26.00 (21.00, 28.50)	0.53
SAS	1.32	38.27 ± 7.62	36.22 ± 6.06	0.19
SDS	568.00	43.00 (36.00, 48.50)	40.00 (34.00, 44.00)	0.06
PSQI	689.50	6.00 (3.00, 9.00)	5.00 (3.00, 8.00)	0.49
Smoking, *n* (%)	1.69	18 (48.6%)	14 (34.1%)	0.19
Drinking, *n* (%)	1.67	13 (35.1%)	9 (22.0%)	0.20
Hypertension, *n* (%)	5.70	27 (73.0%)	19 (41.3%)	0.02*
Hyperlipidemia, *n* (%)	2.36	19 (51.4%)	14 (34.1%)	0.13
Diabetes, *n* (%)	5.29	15 (40.5%)	7 (17.1%)	0.02*
Coronary disease, *n* (%)	0.19	5 (13.5%)	7 (17.1%)	0.66
HDL	/	1.16 ± 0.28	/	/
Homocysteine	/	13.14 ± 7.56	/	/
CP Volume	650.00	2.04 (1.62, 2.32)	1.91 (1.56, 2.21)	0.28
DTI-ALPS	3.78	1.37 ± 0.16	1.50 ± 0.14	**<0.001**
ro-ALPS	4.16	1.37 ± 0.14	1.50 ± 0.14	**<0.001**

MCA-S, middle cerebral artery stenosis; HC, healthy control; BMI, body mass index; TIV; total intracranial volume; MMSE, mini mental state examination; MoCA, Montreal cognitive assessment; SAS, self-rating anxiety scale; SDS, self-rating depression scale; PSQI, Pittsburgh sleep quality index; HDL, high-density lipoprotein; HCY, homocysteine; CP, choroid plexus; DTI-ALPS, diffusion tensor imaging along the perivascular space; ro-ALPS, reoriented diffusion tensor imaging along the perivascular space. Bold values indicate statistical significance (*p* < 0.05). Asterisks (*) indicates statistical significance (*p* < 0.05).

### The differences in ALPS indices between the two groups

**Figure 3:**
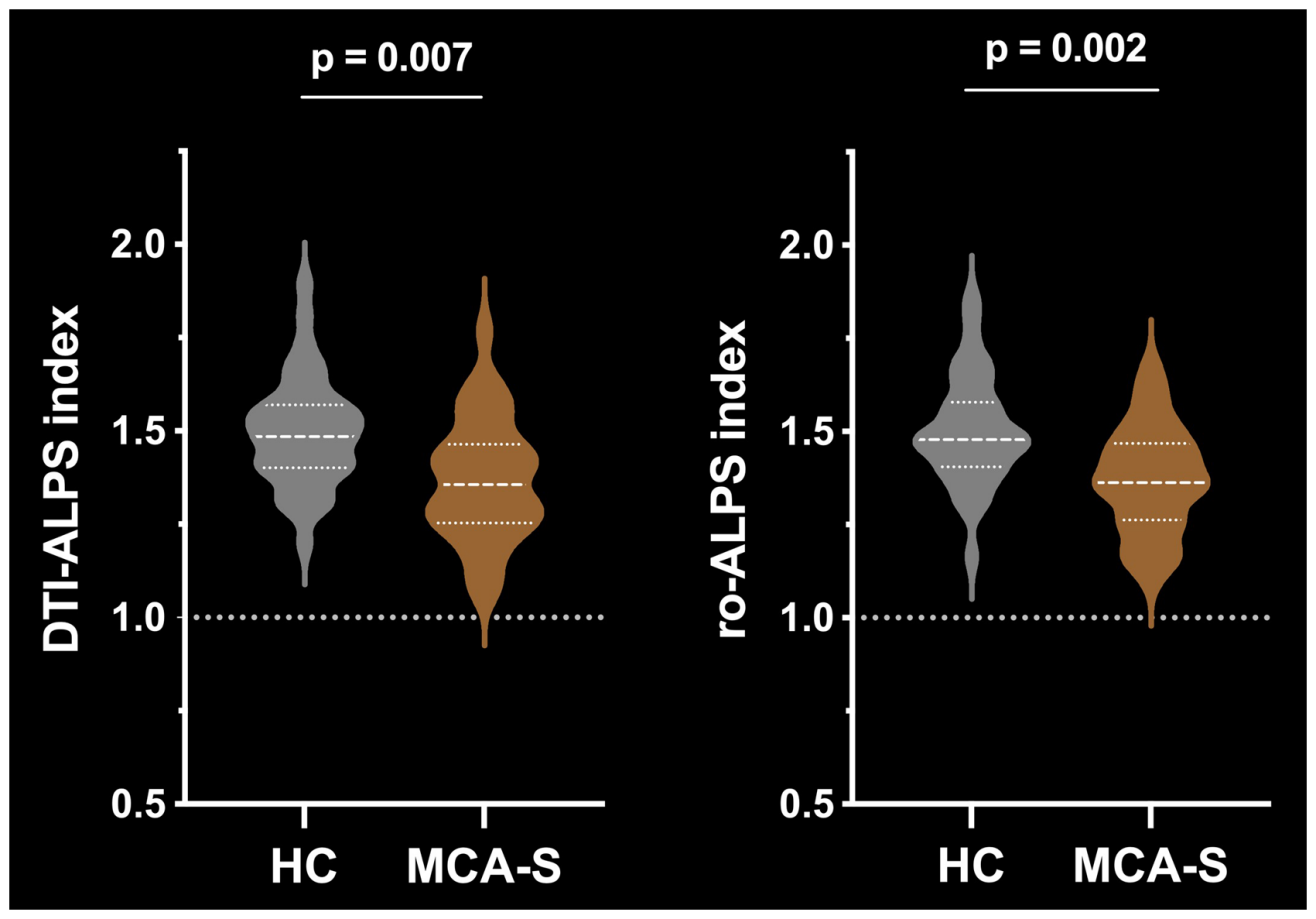
The differences in ALPS indices between healthy controls and MCA-S patients. Between-group differences in diffusion tensor imaging analysis along the perivascular space indices: **(A)** violin plots of the mean DTI-ALPS index in HCs and MCA-S patients; **(B)** violin plots of the mean ro-ALPS index in HCs and MCA-S patients.

The results of the two-sample *t*-test showed that the mean DTI-ALPS index was significantly lower in MCA-S patients than in controls (*t* = 3.78, Cohen’s *d* = 0. 857, *p* < 0.001). Similarly, the re-directed DTI-roALPS index was also significantly lower in the patient group (*t* = 4.16, Cohen’s *d* = 0.945, *p* < 0.001). After adjusting for these covariates, the reduction in the DTI-ALPS index in MCA-S patients remained statistically significant (*F* = 7.78, *p* = 0.007). Similarly, for the ro-ALPS index, the reduction in MCA-S patients persisted after multivariate adjustment (*F* = 9.92, *p* = 0.002). These findings indicate that reduced ALPS indices are associated with MCA-S status and are not solely attributable to demographic differences or vascular comorbidities (Figure 3).

### Correlation analysis of ALPS indices with CP volume, neuropsychological scores, and clinical characteristics

**Figure 4:**
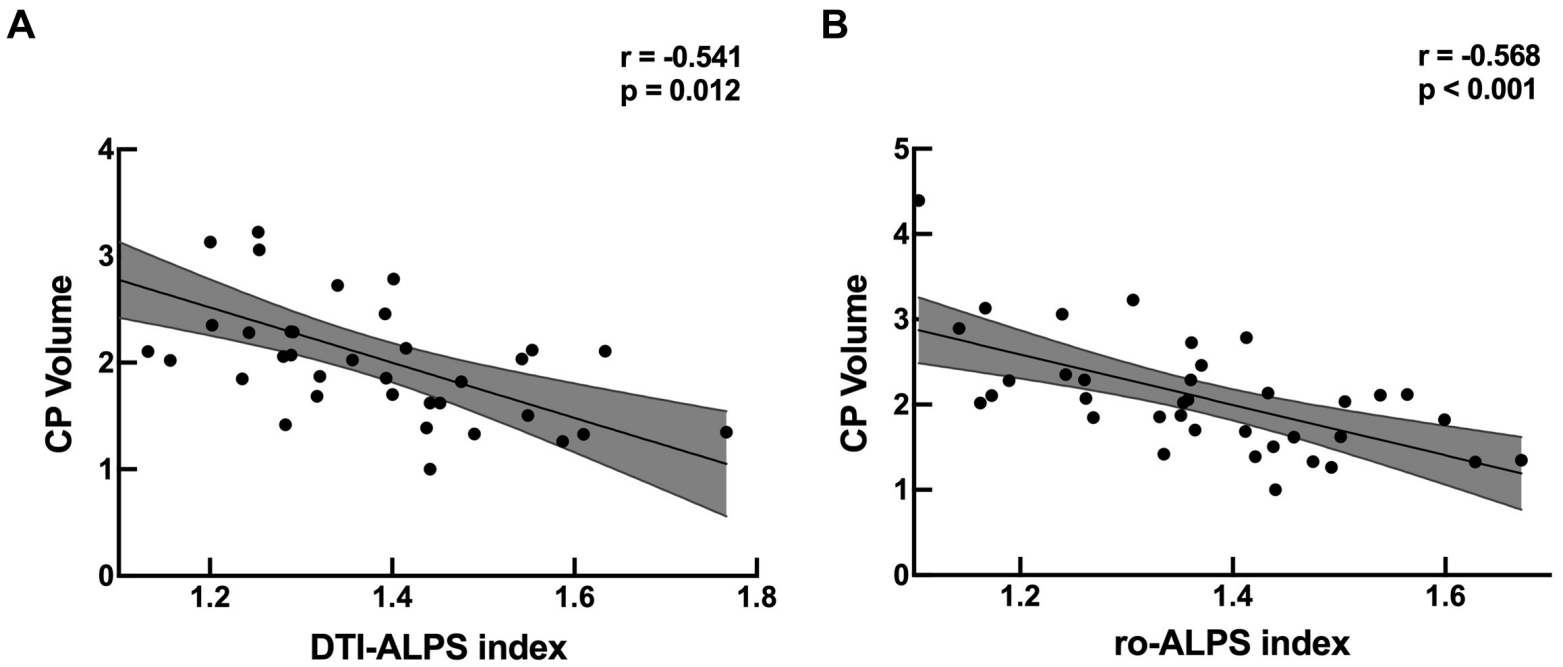
Correlation between ALPS indices and CP volume. **(A)** Correlation between the DTI-ALPS index and CP volume. **(B)** Correlation between the ro-ALPS index and CP volume. DTI-ALPS, diffusion tensor imaging along the perivascular space; ro-ALPS, reoriented diffusion tensor imaging along the perivascular space; CHP/TIV, choroid plexus volume.

Correlation analyses between ALPS indices and clinical indicators, neurocognitive scales and CP volume are shown in Supplementary Tables S2, S3. In the MCA-S group, the DTI-ALPS index was significantly negatively correlated with CP volume (*r* = −0.541, *p* = 0.001, *p_FDR_* = 0.012) (Figure 4). Before FDR correction, the DTI-ALPS index was positively correlated with HDL (*r* = 0.346, *p* = 0.036, *p_FDR_* = 0.108). The ro-ALPS index was also significantly negatively correlated with CP volume (*r* = −0.568, *p* < 0.001, *p_FDR_* < 0.001) (Figure 4). Before FDR correction, the ro-ALPS index was positively correlated with MMSE score (*r* = 0.333, *p* = 0.044, *p_FDR_* = 0.120) and HDL (*r* = 0.337, *p* = 0.041, *p_FDR_* = 0.120), and negatively correlated with homocysteine (*r* = −0.416, *p =* 0.010, *p_FDR_* = 0.060) (Supplementary Table S2). These associations, except for CP volume, did not remain significant after FDR correction. In the HC group, the DTI-ALPS index was significantly negatively correlated with CP volume (*r* = −0.501, *p* < 0.001, *p_FDR_ =* 0.006). The ro-ALPS index was also significantly negatively correlated with CP volume (*r* = −0.508, *p* = 0.001, *p_FDR_* = 0.006) (Supplementary Table S3).

## Discussion

This study jointly evaluated the DTI-ALPS and ro-ALPS indices in patients with MCA-S. The main findings were that both indices were significantly reduced in MCA-S patients compared with HCs and that both indices were negatively correlated with CP volume. Overall, the DTI-ALPS index and the ro-ALPS index show potential as imaging biomarkers for assessing perivascular clearance impairment in MCA-S patients.

In this study, patients with MCA-S exhibited significantly lower DTI-ALPS and ro-ALPS indices than HCs, consistent with the observation that reduced cerebral arterial pulsatility is associated with decreased perivascular clearance in other vascular pathologies (35). We also found a robust negative correlation between both indices and CP volume, present in both MCA-S patients and HCs alike. This suggests that the relationship between perivascular clearance and CP volume may reflect a fundamental physiological characteristic. However, given that MCA-S patients showed lower ALPS indices and larger CP volume than HCs, chronic ischemia may involve pathological structural remodeling (CP enlargement) alongside functional decline. Previous studies have proposed that CP enlargement might mechanically impede CSF turnover by reducing secretion rates (36). We interpret this correlation cautiously, given the cross-sectional nature of our study. Rather than implying a direct causal role in which CP enlargement drives perivascular clearance impairment, both phenomena may arise from shared pathological mechanisms, such as chronic neuroinflammation and vascular comorbidities. First, systemic inflammation and vascular risk factors (e.g., hypertension and diabetes, which were highly prevalent in our MCA-S cohort) can trigger immune cell infiltration and stromal edema in the CP, leading to its enlargement. Simultaneously, these inflammatory processes are known to involve astrocytic reactivity and loss of AQP4 polarization, which are linked to impaired perivascular clearance (37). Second, age-related vascular remodeling and hemodynamic stress from MCA-S may reduce arterial pulsatility (38). This loss of driving force may be associated with reduced perivascular fluid flow and could also compromise the blood-CSF barrier within the CP (39, 40). Future longitudinal studies are needed to clarify the temporal sequence of these structural and functional alterations.

We also explored the relationships of the DTI-ALPS and ro-ALPS indices with systemic vascular risk factors (HDL and homocysteine) and cognitive function (MMSE). Correlation analyses suggested that better vascular health profiles and higher cognitive scores may be associated with higher perivascular diffusivity. However, these associations did not remain significant after FDR correction, likely because of limited statistical power due to the modest sample size.

In addition to the DTI-ALPS index, this study examined the ro-ALPS index. The DTI-ALPS index is a simple measure but requires correct vector directions along the x-, y-, and z axes relative to the brain. Previous studies have shown that changes in imaging plane and head position can affect the reproducibility of the DTI-ALPS index (44). To address this issue, we included the ro-ALPS index in our experimental design. For ro-ALPS index calculation, diffusivity maps with reoriented x-, y-, and z-axes are first created. The reoriented diffusivity maps are appropriately aligned with head position, resulting in small variance in ro-ALPS values and excellent intra- and inter-rater reliability even with slight head rotation. The ro-ALPS index has been used in many recent studies (21, 41–43). In the present study, ro-ALPS showed a slightly larger effect size than the conventional DTI-ALPS index (Cohen’s *d* = 0.945 vs. 0.857). Thus, the current data support the complementary use of both indices rather than definitive superiority of ro-ALPS.

Our study has several limitations. First, the relatively modest sample size limited our statistical power. This may explain why potential associations of the DTI-ALPS index and the ro-ALPS index with systemic vascular risk factors such as HDL and homocysteine did not remain significant after multiple comparison correction. In the between-group comparisons, we adjusted for age, sex, education, and vascular risk factors, and the results remained significant. However, in the correlation analyses, these factors were not adjusted because of the limited sample size, to avoid overfitting and to prevent loss of statistical power. Future studies with larger samples are needed for validation. Second, although the DTI-ALPS index and the ro-ALPS index are widely used surrogate markers, they assess perivascular diffusivity rather than direct tracer-based fluid clearance. Future studies incorporating multimodal imaging, such as IVIM or gBOLD-CSF, could provide a more comprehensive assessment of perivascular clearance dynamics. Third, the cross-sectional design limits inference regarding the temporal sequence of structural changes and functional decline. Longitudinal follow-up is needed to determine whether these changes represent sequential events or parallel consequences of shared vascular pathology.

## Conclusion

In summary, these findings support the potential utility of ALPS indices as non-invasive imaging biomarkers for characterizing perivascular clearance alterations associated with MCA-S.

## Data Availability

The raw data supporting the conclusions of this article will be made available by the authors, without undue reservation.

## Footnotes

1
https://identifiers.org/neurovault.image:1408
