# Supplementary material for: Non-invasive assessment of glymphatic dysfunction in middle cerebral artery stenosis based on DTI-ALPS and ro-ALPS
Source: Front Neurol. 2026 Jun 17;17:1826663. doi: 10.3389/fneur.2026.1826663 (PMC13318568; doi:10.3389/fneur.2026.1826663)
Supplement: Supplementary file 1 [file Table_1.DOCX]

**Supplementary Table S1 Results of the Shapiro-Wilk normality test for all continuous variables.**

|  | Group | Shapiro-Wilk (W) | *P*-value |
| --- | --- | --- | --- |
| Age(years) | HC | 0.906 | 0.003 |
|  | MCA-S | 0.938 | 0.041 |
| Education(years) | HC | 0.956 | **0.115** |
|  | MCA-S | 0.916 | 0.009 |
| BMI | HC | 0.940 | 0.033 |
|  | MCA-S | 0.973 | **0.508** |
| TIV | HC | 0.972 | **0.394** |
|  | MCA-S | 0.979 | **0.710** |
| MMSE | HC | 0.811 | <0.001 |
|  | MCA-S | 0.749 | <0.001 |
| MoCA | HC | 0.877 | <0.001 |
|  | MCA-S | 0.832 | <0.001 |
| SAS | HC | 0.963 | **0.205** |
|  | MCA-S | 0.965 | **0.291** |
| SDS | HC | 0.955 | **0.107** |
|  | MCA-S | 0.927 | 0.019 |
| PSQI | HC | 0.937 | 0.025 |
|  | MCA-S | 0.905 | 0.004 |
| CP Volume | HC | 0.965 | **0.230** |
|  | MCA-S | 0.920 | 0.011 |
| DTI-ALPS | HC | 0.978 | **0.608** |
|  | MCA-S | 0.986 | **0.924** |
| ro-ALPS | HC  MCA-S | 0.976  0.983 | **0.523**  **0.834** |

MCA-S, Middle cerebral artery stenosis; HC, Healthy control; BMI, Body mass index; TIV; **Total intracranial volume**; MMSE, Mini-mental state examination; MoCA, Montreal cognitive assessment; SAS, Self rating anxiety scale; SDS, Self rating depression scale; PSQI, Pittsburgh sleep quality index; CP, Choroid plexus; DTI-ALPS, **diffusion Tensor Imaging along the Perivascular Space;** ro-ALPS, **Reoriented diffusion tensor imaging along the perivascular space**
