# Supplementary material for: Non-invasive assessment of glymphatic dysfunction in middle cerebral artery stenosis based on DTI-ALPS and ro-ALPS
Source: Front Neurol. 2026 Jun 17;17:1826663. doi: 10.3389/fneur.2026.1826663 (PMC13318568; doi:10.3389/fneur.2026.1826663)
Supplement: Supplementary file 2 [file Table_2.DOCX]

**Supplementary Table S2. Correlation between the DTI-ALPS index, Clinical indicators and neurocognitive scales in MCA-S**

|  |  | | | DTI-ALPS index | | ro-ALPS | |  |
| --- | --- | --- | --- | --- | --- | --- | --- | --- |
|  | *r* | | *p* value | | *p_FDR_* | *r* | *p* value | *p_FDR_* |
| MoCA | | 0.356 | 0.031 | | 0.108 | 0.325 | 0.050 | 0.120 |
| MMSE | | 0.298 | 0.073 | | 0.880 | 0.333 | 0.044 | 0.120 |
| SAS | | 0.114 | 0.503 | | 0.862 | 0.054 | 0.753 | 0.904 |
| SDS | | 0.032 | 0.850 | | 0.948 | -0.044 | 0.797 | 0.904 |
| PSQI | | -0.026 | 0.878 | | 0.948 | -0.057 | 0.739 | 0.904 |
| CP | | -0.541 | 0.001 | | 0.012** | -0.568 | <0.001 | <0.001** |
| HCY | | -0.365 | 0.026 | | 0.108 | -0.416 | 0.010 | 0.060 |
| HDL | | 0.346 | 0.036 | | 0.108 | 0.337 | 0.041 | 0.120 |
| ApoA-1 | | 0.146 | 0.390 | | 0.780 | 0.107 | 0.529 | 0.904 |
| ApoB | | 0.081 | 0.634 | | 0.948 | 0.037 | 0.829 | 0.904 |
| LDL | | 0.048 | 0.777 | | 0.948 | 0.020 | 0.908 | 0.908 |
| TG | | -0.011 | 0.948 | | 0.948 | -0.041 | 0.809 | 0.904 |

* Indicate statistically significant *p* < 0.05. ** indicates statistically significant *p* < 0.05 after FDR correction. MMSE, mini-mental state examination; MoCA, Montreal cognitive assessment; PSQI, Pittsburgh sleep quality index; SAS, Self rating anxiety scale; SDS, Self rating depression scale; CP, Choroid plexus; HCY, homocysteine; HDL, High-density lipoprotein; ApoA-1, Apolipoprotein A1; ApoB, Apolipoprotein B; LDL, Low-density lipoprotein; TG, Triglycerides.
