# Supplementary material for: Non-invasive assessment of glymphatic dysfunction in middle cerebral artery stenosis based on DTI-ALPS and ro-ALPS
Source: Front Neurol. 2026 Jun 17;17:1826663. doi: 10.3389/fneur.2026.1826663 (PMC13318568; doi:10.3389/fneur.2026.1826663)
Supplement: Supplementary file 3 [file Table_3.DOCX]

**Supplementary Table S3. Correlation between the DTI-ALPS index, Clinical indicators and neurocognitive scales in HC**

|  |  | | DTI-ALPS index | |  | ro-ALPS | |
| --- | --- | --- | --- | --- | --- | --- | --- |
|  | *r* | *p* value | | *p_FDR_* | *r* | *p* value | *p_FDR_* |
| MoCA | 0.225 | 0.157 | | 0.314 | 0.267 | 0.091 | 0.182 |
| MMSE  SAS  SDS  PSQI  CP | 0.301  -0.182  -0.050  -0.069  -0.501 | 0.056  0.255  0.758  0.670  0.001 | | 0.168  0.383  0.758  0.758  0.006** | 0.301  -0.172  -0.043  -0.080  -0.508 | 0.056  0.282  0.789  0.618  0.001 | 0.168  0.423  0.789  0.742  0.006** |

*Indicate statistically significant *p* < 0.05. ** indicates statistically significant *p* < 0.05 after FDR correction. MMSE, mini-mental state examination; MoCA, Montreal cognitive assessment; PSQI, Pittsburgh sleep quality index; SAS, Self rating anxiety scale; SDS, Self rating depression scale; CP, choroid plexus.
